# Effects of doxycycline dose rate and pre-adulticide wait period on heartworm-associated pathology and adult worm mass

**DOI:** 10.1186/s13071-023-05858-2

**Published:** 2023-07-25

**Authors:** Andrew R. Moorhead, Christopher C. Evans, Kaori Sakamoto, Michael T. Dzimianski, Abdelmoneim Mansour, Utami DiCosty, Crystal Fricks, Scott McCall, Ben Carson, C. Thomas Nelson, John W. McCall

**Affiliations:** 1 Department of Infectious Diseases, College of Veterinary Medicine, University of Georgia, Athens, GA USA; 2 Department of Pathology, College of Veterinary Medicine, University of Georgia, Athens, GA USA; 3 TRS Labs Inc., P.O. Box 5112, Athens, GA 30607 USA; 4 Animal Medical Center, 719 Quintard Ave, Anniston, AL 30605 USA

**Keywords:** Heartworm, *Dirofilaria immitis*, Dog, Doxycycline, Pathology, Adulticide

## Abstract

**Background:**

The American Heartworm Society canine guidelines recommend treatment with doxycycline prior to adulticide administration to reduce levels of *Wolbachia* and its associated metabolites, which are known to be a leading cause of pulmonary pathology. Studies have determined that doxycycline administered at 10 mg/kg BID for 28 days is an effective dose for eliminating *Wolbachia*, but what has not been determined is the clinical relevance of this elimination. The current guidelines also recommend a 30-day wait period following administration of doxycycline to allow for clearance of metabolites, such as *Wolbachia* surface protein, and for further reduction in heartworm biomass before administration of adulticide. Reducing the doxycycline dose and eliminating the wait period may carry practical benefits for the animal, client, and practitioner.

**Methods:**

To investigate these treatment practices, *Dirofilaria immitis* adults were surgically transplanted into each of 45 dogs, which were divided into nine study groups of five dogs each. Seventy-five days after transplantation, two groups each were administered 5, 7.5, or 10 mg/kg BID doxycycline orally for 28 days and 6 µg/kg ivermectin monthly, with three untreated groups serving as controls. Study animals were necropsied and examined prior to treatment as well as 30 and 60 days post-treatment.

**Results:**

Mean worm weight was unaffected by dosage but exhibited a significant increase at 30 days and significant decrease at 60 days post-treatment, including in control groups. Histopathology lesion scores did not significantly differ among groups, with the exception of the lung composite score for one untreated group. Liver enzymes, the levels of which are a concern in doxycycline treatment, were also examined, with no abnormalities in alanine aminotransferase or alkaline phosphatase observed.

**Conclusions:**

No consistent worsening of tissue lesions was observed with or without the AHS-recommended 30-day wait period, nor did reduced dosages of doxycycline lead to worsening of pathology or any change in efficacy in depleting worm weight. Mean worm weight did significantly increase prior to, and decrease following, the wait period. Future work that also includes adulticide treatment (i.e. melarsomine) will study treatment recommendations that may improve both animal health and owner compliance.

**Graphical abstract:**

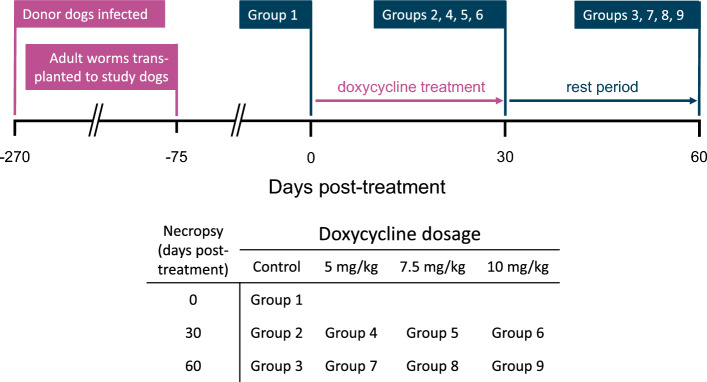

**Supplementary Information:**

The online version contains supplementary material available at 10.1186/s13071-023-05858-2.

## Background

The American Heartworm Society (AHS)-recommended treatment protocol for canine heartworm disease specifies the use of melarsomine dihydrochloride, and prior to injection of this drug, administration of an approved macrocyclic lactone (ML) is recommended. Concomitant with the first dose of ML, it is also recommended that doxycycline (10 mg/kg BID) be administered for 4 weeks, specifically to adversely affect the filarial endosymbiont *Wolbachia*. The benefits of this administration of ML and doxycycline have been reported by multiple authors. Bazzocchi et al. [1] reported that the combination of ivermectin and doxycycline affected the viability of both microfilariae and adult heartworms. When weekly prophylactic doses of ivermectin (6 µg/kg) were administered orally for 36 weeks and doxycycline (10 mg/kg/day) was given orally from weeks 1‒6, 10‒12, 16‒18, 22‒26, and 28‒34, the number of adult heartworms were significantly reduced compared to controls. A reduction of microfilariae after 6 weeks and elimination of microfilariae after 12 weeks was observed. Furthermore, in doxycycline-treated dogs, adult worms experienced significant decreases in *Wolbachia* DNA copy numbers [1]. This study also showed that administration of doxycycline in combination with ivermectin provided more rapid adulticidal activity than ivermectin alone and reduced *Wolbachia* numbers more effectively than doxycycline alone [1]. More recently, it has been shown that oral administration of doxycycline at 10 mg/kg SID for 30 days and ivermectin at 6 µg/kg on days 0 and 30 (short-treatment regimen group) eliminated circulating microfilariae by day 239 and reduced adult worm count by 57.5% at necropsy on day 647 [2]. In the same study, oral administration of doxycycline at the same dosage for 72–98 days and ivermectin at the same dosage for 6–7 biweekly doses (long-treatment regimen group) terminated circulating microfilariae by day 97 and reduced adult worm count by 79.3% at necropsy. In that study gender bias effect was noted, with all of the female worms removed in the long-treatment regimen group and most of the females killed in the short-treatment regimen group. Moxidectin, when coupled with doxycycline, has been shown to have adulticidal activity [3, 4].

While the effects of doxycycline and MLs on heartworm have been documented, studies have also examined the effect on the host. One such study showed that experimentally infected heartworm-positive dogs pretreated with ivermectin and doxycycline prior to melarsomine injection had less severe pulmonary lesions associated with the death of the heartworms [5, 6]. A retrospective study of clinical cases reported a 65% decrease in respiratory complications in dogs treated with monthly prophylactic doses of ivermectin (6 µg/kg) and doxycycline (10 mg/kg BID for 28 days) that were then treated with the three-dose melarsomine protocol after a 30-day rest period [7, 8]. No studies have been published on the combination of milbemycin oxime or selamectin with doxycycline.

Two of the major persistent questions regarding the current AHS-recommended treatment protocol are whether the dose of doxycycline can be reduced and whether a month waiting period between the end of doxycycline administration and the first dose of melarsomine dihydrochloride is beneficial. Reduction in the recommended doxycycline dosage for canine heartworm disease from the current 10 mg/kg BID to 5 mg/kg BID has been proposed by Carretón et al. [9] based on a study that measured anti-rWSP antibodies. The authors of this study suggested that a lower dose of doxycycline was sufficient to reduce *Wolbachia* levels in *Dirofilaria immitis* based on decreased antibody levels. This study did not report the rate of post-treatment complications, nor were necropsies performed to assess pulmonary pathology. Another clinical study performed by Savadelis et al. [10] measured the levels of *Wolbachia* in microfilariae in dogs that were treated using the AHS treatment protocol. These dogs received a prophylactic dose of ivermectin (6 µg/kg) followed by a 28-day course of minocycline or doxycycline at a dose of 5 mg/kg or 10 mg/kg BID. Thirty days after the completion of antibiotics, dogs received the standard three-dose melarsomine dihydrochloride regimen. When *Wolbachia* DNA levels of circulating microfilariae were assessed, only the 10 mg/kg BID doxycycline group fell below the limit of detection. Bowman and Drake [11] have advocated for the elimination of the entire pretreatment and rest phases, as delaying melarsomine therapy has been hypothesized to lead to a significant increase in heartworm biomass and pulmonary pathology. Furthermore, it has been suggested that the delay of melarsomine treatment puts an increased burden on owners as well as shelter and rescue groups. Carretón et al. have suggested that eliminating the month-long wait between doxycycline and melarsomine administration may be beneficial as it shortens the time to elimination of adult heartworms and may increase owner adherence to the recommended treatment protocol [9].

Doxycycline treatment has also been noted to have an effect on liver enzyme levels. It has been reported that both alanine aminotransferase (ALT) and alkaline phosphatase (ALP) were increased in 39.4% and 36.4% of dogs (*n* = 386) administered doxycycline, respectively [12]. Concern has been expressed anecdotally that the use of doxycycline in heartworm treatment could contribute to an increase in liver enzymes and other signs associated with doxycycline administration. Examining the rate and severity of enzyme level increase could clarify the validity of this concern.

The purpose of this study was to determine the optimal dose of doxycycline for the standard treatment of canine heartworm disease and to determine whether the rest phase after doxycycline treatment is beneficial. Our first objective was to determine whether administration of doxycycline for 28 days (per the AHS guidelines) at 5, 7.5, or 10 mg/kg BID when combined with monthly ivermectin resulted in significant differences in pulmonary, hepatic, and renal pathology between different dosages and between 30 and 60 days after the beginning of treatment. By examining the pathology at these time points, we also assessed whether the current 30-day rest period between the end of doxycycline therapy and the beginning of melarsomine injections could potentially result in worsening or improved pathology. Also, at 30 and 60 days post-treatment, we examined worm biomass at an individual worm level to assess whether the combined administration of ivermectin and doxycycline results in reduced heartworm biomass.

## Methods

### Study animals

**Fig. 1:**
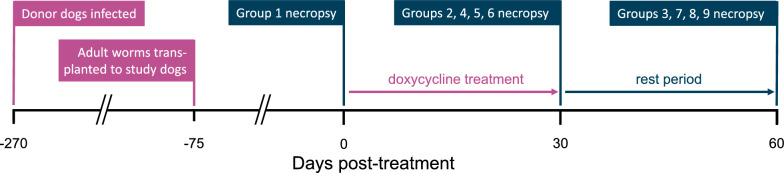
Study timeline

Fifteen purpose-bred, specific-pathogen-free dogs were experimentally infected with heartworm (*D. immitis*) by injecting approximately 250 third-stage infective larvae subcutaneously into the flank. Adult worms were collected 6.5 months post-infection, at which time 8 male and 12 female adult heartworms were individually weighed and introduced by intravenous transplantation into 45 young adult dogs. Recipient dogs were randomly divided into nine groups of five dogs each (Table 1). Groups 1, 2, and 3 served as non-treated controls. Treatment groups were treated with 6 µg/kg ivermectin monthly and one of three doses of doxycycline for 28 days, starting at 75 days post-transplantation (treatment day 0): Groups 4 and 7 received 5 mg/kg BID doxycycline; Groups 5 and 8 received 7.5 mg/kg BID doxycycline; Groups 6 and 9 received 10 mg/kg BID doxycycline. On treatment day 0 (Fig. 1), control Group 1 was killed and necropsied, and worms were collected, counted, and individually weighed. Following worm removal, tissue samples were collected for histopathology as described in the following section. On treatment day 30, Groups 2, 4, 5, and 6 were killed and necropsied, and worms and tissues were collected in the same manner. On treatment day 60, Groups 3, 7, 8, and 9 were killed, necropsied, and processed in the same manner. Dogs in study groups had blood drawn weekly for serum chemistries and microfilaria counts.

**Table 1:** Experimental groups for doxycycline treatment regimens

Necropsy (days post-treatment)	Doxycycline dosage			
	Control	5 mg/kg	7.5 mg/kg	10 mg/kg
0	Group 1			
30	Group 2	Group 4	Group 5	Group 6
60	Group 3	Group 7	Group 8	Group 9

Dogs treated with doxycycline also received 6 µg/kg ivermectin monthly starting at day 0

### Worm weight assessment

Heartworms harvested from donor dogs were placed in Hanks’ balanced salt solution (HBSS) with penicillin (100 U/ml) and streptomycin (100 mg/ml). Heartworms were divided into 45 groups of 20 worms each consisting of 12 female and 8 male worms. Worms were removed from HBBS and placed on a sterile surgical towel for 1 min to absorb excess solution. Individual worms were weighed and then placed in a stainless-steel bowl containing 100 ml HBSS that had been previously weighed. The bowl containing the worms was again weighed, and the combined weight of the 20 heartworms was recorded. Worms collected from study animals during necropsy were weighed individually in the same manner.

### Microfilaria and antigen testing

EDTA-anticoagulated blood was examined by modified Knott test for the presence of microfilariae.

Antigen testing was performed monthly on serum collected from whole blood using the DiroCHEK^®^ Heartworm Antigen Test Kit (Zoetis, Florham Park, NJ). The DiroCHEK^®^ test was performed according to the manufacturer’s recommendations using undiluted serum, both heat-treated and not heat-treated. Heat-treatment was performed using a dry heat block at 103 °C for 10 min followed by centrifugation. Antigen levels were determined using a spectrophotometer at a wavelength of 490 nm and analyzed according to positive and negative controls provided with the kit.

### Tissue histology

At necropsy, samples from the right caudal lung lobe (as well as any grossly noted lesions) and representative sections from the liver, kidney, right and left ventricles, and pulmonary artery of each dog were fixed in 10% neutral-buffered formalin, processed for routine histology, and stained with hematoxylin and eosin. Samples were evaluated for lesions and assigned a lesion score (0–4) to each tissue section according to the criteria described in Table 2 by a board-certified veterinary pathologist blinded to the treatment groups. For evaluating changes between time points and doses, composite scores were calculated as the sum of relevant individual scores for lung (thrombi, endarteritis, fibrosis, and vascular proliferation scores), kidney (cortical and medullary interstitial nephritis and renal interstitial fibrosis scores), and liver tissue (necrosis, granulomas, and portal/caudate lobe scores).

**Table 2:** Lesion scoring criteria for tissue histology in dogs experimentally infected with *Dirofilaria immitis*

Tissue		Score			
		1	2	3	4
Heart	Pulmonary artery	Rare, small, intimal proliferation	Multifocal, regular proliferation	Multifocal, villous proliferation	Multifocal bulbous to plaque-like proliferation
Lung	Thrombus	Vessel < 25% occluded	Vessel 25–49% occluded	Vessel 50–74% occluded	Vessel > 75% occluded
	Endoarteritis	WBC infiltration limited to intima	Up to 50% vessel wall	50–99% infiltration	Transmural
	Fibrosis	Focal	Multifocal	Locally extensive	Diffuse
	Vascular proliferation	Focal	Multifocal	Locally extensive	Diffuse
Liver	Necrosis	Mild	Moderate	Marked	N/A
	Granuloma	Low numbers of leukocytes	Moderate numbers of leukocytes	Large numbers of leukocytes	N/A
	Portal/centrilobular infiltrates	Low numbers of leukocytes	Moderate numbers of leukocytes	Large numbers of leukocytes	N/A
Kidney	Interstitial nephritis (cortex)	Rare, minimal infiltrates	Multifocal, ≤ 10 cells	Multifocal, > 10 cells	Multifocal to coalescing w/ necrosis
	Interstitial nephritis (medulla)	Rare, minimal infiltrates	Multifocal, ≤ 10 cells	Multifocal, > 10 cells	Multifocal to coalescing w/ necrosis
	Interstitial fibrosis/ glomerulosclerosis	Mild, focal, one or the other	Multifocal, mild in both	Multifocal, moderate in both	Locally extensive in one or both

### Serum chemistries

Blood samples were obtained from all study dogs on day − 1 prior to any treatment. Starting on treatment day 14, weekly blood samples were collected from control and treated dogs until they were killed. Serum was separated, placed in plastic tubes, and frozen at − 20 °C. All samples were submitted to a reference laboratory (Zoetis Reference Laboratories) for a comprehensive blood chemistry panel.

### Data analysis

Mean individual weight of worms at the time of transplantation and time of recovery was used to calculate a percentage increase or decrease at each study time point. Comparisons between different doses of doxycycline as well as before and after the 30-day rest period were performed by two-way ANOVA with time and dose as factors. The Tukey test was used for post hoc comparisons.

Lung lesion scores were used to assess whether lower doses of doxycycline resulted in a non-inferior reduction in pulmonary pathology compared to the current standard dose of 10 mg/kg BID for 28 days. Lung lesion scores between treatment day 30 and day 60 and between the different doxycycline doses groups were compared by two-way ANOVA with time and dose as factors. The Bonferroni test was performed for post hoc comparisons of time points, while the Tukey test was performed for post hoc comparisons between doses.

All analyses were performed in Graphpad Prism 8.0 (GraphPad Software, La Jolla, CA) with familywise α = 0.05.

## Results

### Microfilariae and antigen

All study animals developed detectable microfilaremia and antigenemia. By the end of the study, microfilaremia fell below detectable levels in 3 of 20 dogs (2 in the 5 mg/kg doxycycline group and 1 in the 7.5 mg/kg doxycycline group, all necropsied at the 60 day post-treatment time point).

### Worm weight

**Fig. 2:**
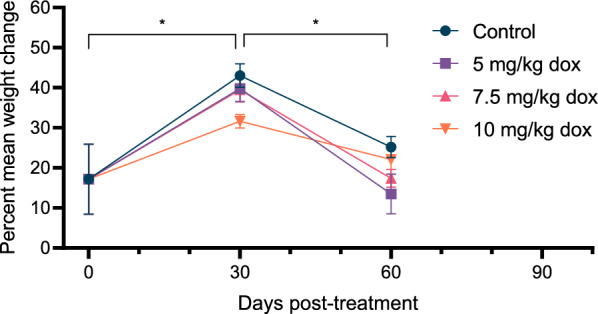
Mean weight of individual heartworms for each doxycycline treatment group at each time point examined. Mean worm weights were calculated for all worms recovered per study animal within each group. Asterisks indicate significant differences between time points within all study groups (*p* < 0.05)

Mean weight per recovered worm was calculated with comparisons made between dose groups and time points (Fig. 2, Additional file 2: Table S2). For all dose groups, a significant increase in mean weight change was observed from the start of treatment (mean = 17.2%, range = 8.28–30.3%) to 30 days post-treatment (mean = 38.5%, range = 30.2–46.5%; *p* < 0.001). A significance decrease was then observed for all groups at 60 days post-treatment (mean = 19.5%, range = 7.10–28.0%; *p* < 0.028). No statistically significant differences were observed among dose groups at any time point (*p* = 0.069).

### Histopathology

**Fig. 3:**
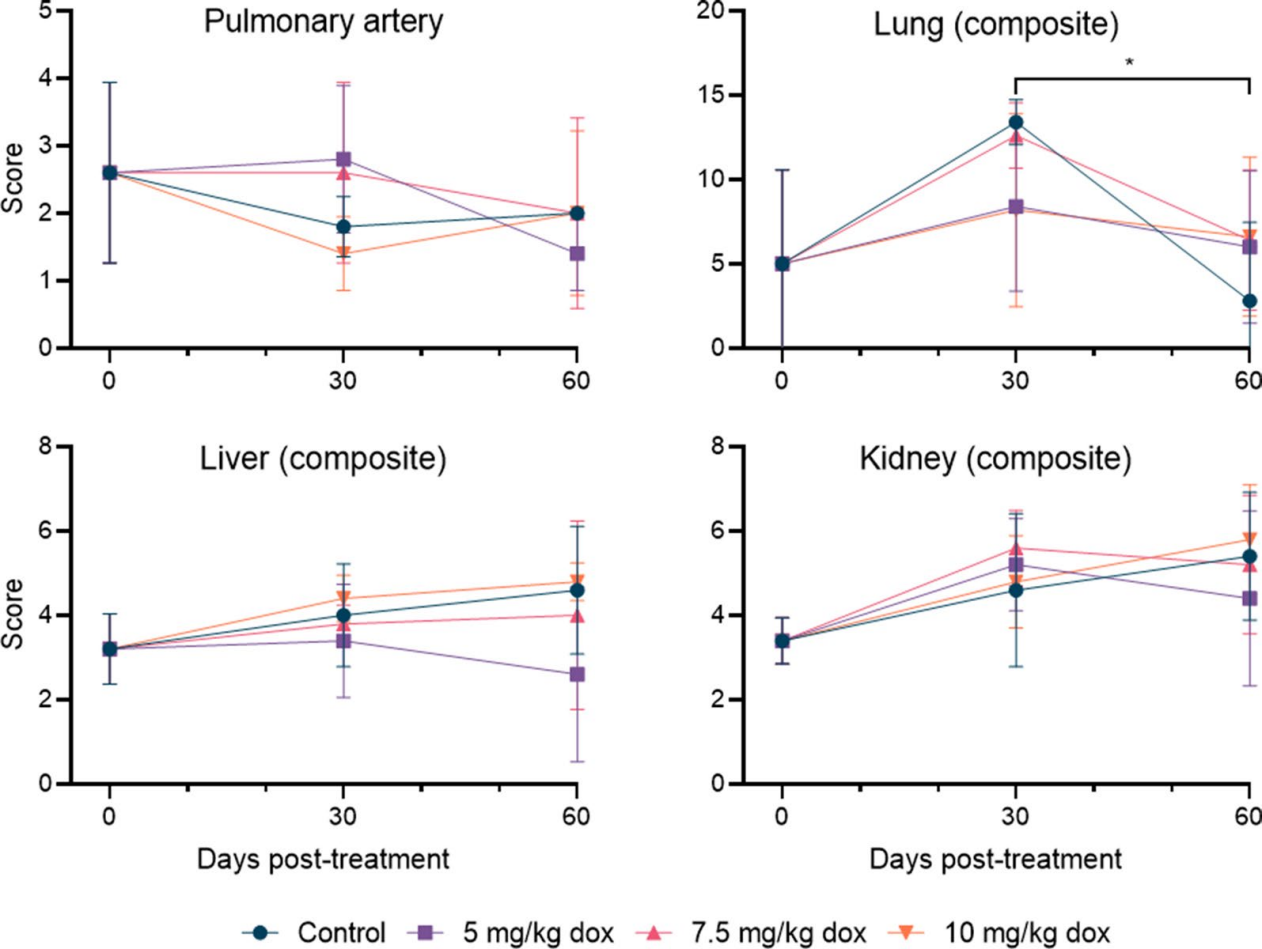
Mean histopathology scores of heartworm-infected dogs for each doxycycline treatment group at each time point examined. All tissues were scored on a scale of 0–4. Composite scores represent the sum of relevant individual scores for each tissue. Asterisk indicates a significant difference between time points within the untreated control group. The only significant difference was between day 30 and day 60 for the control group lung composite score (*p* < 0.0017)

With one exception (control group lung composite score; *p* < 0.0017), no statistically significant differences in individual or composite histopathologic scores were observed between doxycycline dose groups or between time points before and after the month-long rest period (*p* ≥ 0.079; Fig. 3). The mean pulmonary artery score across dose groups exhibited a slight decrease from the start of treatment (2.6, range = 1–4) to 30 days (mean = 2.2, range = 1–4) and 60 days (mean = 1.8, range = 0–4) post-treatment. The mean lung composite score increased from a baseline prior to treatment (5.00, range = 0–14) to 30 days post-treatment (mean = 10.6, range = 0–14) and then decreased at 60 days post-treatment (mean = 5.45, range = 0–12). The mean liver composite score increased slightly from prior to treatment (3.2, range = 2–4) to 30 days (mean = 3.9, range = 2–5) post-treatment, remaining similar at 60 days post-treatment (mean = 4.0, range = 0–6). Finally, the mean kidney composite score increased from prior to treatment (3.4, range = 3–4) to 30 days (mean = 5.1, range = 3–7) post-treatment, again remaining similar at 60 days post-treatment (mean = 5.2, range = 2–8).

### Serum chemistries

Kidney values, glucose, albumin, globulins, and electrolytes were normal in all dogs during the study. The predominant finding was mild elevation (less than twice the upper reference range) of ALT in 10 dogs. Two dogs had moderate elevation in ALT (2–5 times the upper reference range). No dogs in Groups 1 and 2 (control) or Groups 4 and 5 (5 mg/kg and 7.5 mg/kg doxycycline, respectively) had an elevation in ALT at any point. In Group 3 (control) four of five dogs exhibited elevated ALT. One dog in Group 6 (10 mg/kg doxycycline) had a mild elevation of ALT on day − 1 but was normal during the period of doxycycline administration. Two dogs in Group 7 (5 mg/kg doxycycline) had mild elevation of ALT during doxycycline therapy but both returned to normal after completion of treatment. One dog in Group 8 (7.5 mg/kg doxycycline) had mildly elevated ALT at day − 1 and remained elevated, rising to the moderate level at day 42; a second dog in this group had mildly elevated ALT at day 42. Two dogs in Group 9 (10 mg/kg doxycycline) had mildly elevated ALT after doxycycline treatment. One dog in Group 9 had a mildly elevated ALT at day − 1 that dropped to normal levels during doxycycline treatment, again rising to mildly elevated levels after completion of treatment. Elevated ALP levels were never observed in any study dog.

## Discussion

The AHS guidelines recommend three doses of melarsomine preceded by 28 days of doxycycline (10 mg/kg BID) followed by a 28-day waiting period with the hypothesized benefit of a complete elimination of the endosymbiont *Wolbachia* [13]. However, some questions remain surrounding both the optimal dosage of doxycycline and the benefit of the 30-day waiting period. The purpose of this study was to determine whether a reduced dose of doxycycline, along with ivermectin, and removal of the waiting period would result in any change in pathology in the host. We transplanted adult heartworms into dogs, and 75 days later, ivermectin (6 µg/kg PO) and doxycycline (5, 7.5, or 10 mg/kg BID PO) were administered to experimental groups. Animals were killed 30 and 60 days after the start of ivermectin/doxycycline treatment. We then examined the histopathology of the host kidneys, lungs, liver, and pulmonary arteries as well as worm biomass and liver enzyme values. We noted no clinical signs related to heartworm infection during the course of the study. Overall, we observed neither a significant change in histopathological abnormalities nor alteration of liver values between days 30 and 60 of the study, suggesting that neither the dosage nor the additional month wait period affects the host in a positive or negative manner. Also, mean weight per worm significantly increased from study day 0 to 30 and then subsequently significantly decreased from study day 30 to 60 at all doses and the untreated control group. An increase in worm weight from study day 0 to 30 was expected as the worms were 9 months old and reaching the age where the female worms would be producing microfilariae. The decrease in weight from study day 30 to 60 was also expected as *Wolbachia* levels would have been depleted, which would have a negative effect on microfilariae production as well as the health of the adult worms. The decrease in worm weight in the control group between study day 30 and 60 was not expected. In reviewing the data, it was noted that the day 30 and day 60 control groups had worm recovery rates of 78.3% and 75%, respectively, compared to the day − 75 control group’s 95%. It has been reported that experimentally transplanted worm recovery rates averaged 91.3% [14]. To obtain worm weight data for comparison, the worms to be transplanted were first measured for length to select similarly sized worms. They were then placed on a dry, sterile towel for 1 min to absorb excess fluid before being individually weighed and then placed in a sterile bowl containing 100 ml HBSS as described in the Methods section. This degree of handling would have been stressful to the worms and could explain the decreased worm recovery. In reviewing the pathology data, it was noted that the thrombi scores were 4 (scale 0–4) in all the dogs in the 30-day control group, and dead worms were noted in 3 of 5 dogs, indicating a notable degree of worm death during the day 0 to day 30 period. The presence of these dead worms would have precipitated the release of inflammatory mediators and a marked immune response [15, 16], which would affect the health of the remaining worms, theoretically leading to a loss of biomass.

The recommended period between diagnosis of heartworm infection and administration of the first melarsomine injection has varied. Macrocyclic lactone preventives will eliminate susceptible third- and fourth-stage larvae, whereas melarsomine will treat adult heartworms. Previously, 2- to 4-month-old heartworms were postulated not to be susceptible to either drug, and a waiting period of 2 to 3 months was initially proposed to allow these worms to reach at least 4 months of age and thus age into susceptibility to melarsomine. This concept of life cycle stages not susceptible to either drug was termed the “treatment gap” or “susceptibility gap.” The incorporation of doxycycline into the first month of the susceptibility gap was logical, as doxycycline administration along with ivermectin had been shown to decrease the number of *Wolbachia* within worms and also to decrease overall worm mass [1]. That study, however, used an extended regimen of doxycycline without melarsomine. Kramer et al. [5] showed that a reduced 30-day course of doxycycline followed by a 2-month waiting period decreased lung pathology in animals receiving melarsomine compared to those receiving melarsomine alone. The AHS shortened the period between diagnosis and melarsomine treatment to 2 months in 2014 [18].

Bowman and Drake postulated that the susceptibility gap was not a concern due to the efficacy of macrocyclic lactones and melarsomine against larval and adult stages in the definitive host. Furthermore, by incorporating a wait period, the worms could increase in size, resulting in an increase in observable pathology [11]. While this is a concern, the benefits of doxycycline, specifically the reduction in lung pathology post-melarsomine, are still included in the AHS guidelines. Furthermore, a 1-month period after doxycycline administration is still included in AHS treatment guidelines to account for the hypothesized possibility of *Wolbachia* metabolites and surface proteins persisting in the worms [18]. These metabolites and surface proteins could cause increased host pathology after melarsomine-induced worm death.

In the current study, we evaluated pathology and worm biomass at both 30 and 60 days after the beginning of treatment. We observed no significant differences in pathology between time points or dosages of doxycycline (5, 7.5, or 10 mg/kg BID) with the exception of a difference in the lungs of the control group between 30 and 60 days. These results suggest that the inclusion of a 30-day waiting period does not affect pathology. We administered no melarsomine during this study, so there is the possibility that we would have observed more pronounced differences between doxycycline treatment groups with the inclusion of melarsomine. While we did not note any apparent clinical signs in the study animals, further observation after melarsomine treatment would provide information on possible complications encountered by veterinarians and owners in the clinical setting compared to the design of the present controlled study. In a clinical study, Carretón et al. [19] evaluated 76 dogs that received 30 days of doxycycline (10 mg/kg BID) followed immediately by a three-dose course of melarsomine. Clinical signs were observed in 60.5% of dogs, but as none received melarsomine 60 days after the start of doxycycline, the impact of the wait month cannot be assessed. While we observed no increase in pathology in the current study, we cannot conclude whether clinical signs will worsen or improve after melarsomine administration at 30 or 60 days in dogs given varying dosages of doxycycline.

In our current study, we observed a significant decrease in individual mean worm weight between days 30 and 60 for all dosages of doxycycline and the untreated control group. These results agree with the observations by Bazzochi et al. [1] that a combination of ivermectin and doxycycline together decrease worm size, presumably because of elimination of *Wolbachia*, though this does not account for the reduction in worm weight observed in the control group. In a clinical study of dogs treated with differing dosages of doxycycline and minocycline, *Wolbachia* was shown to be eliminated as assessed by qPCR from microfilariae after a 28-day regimen of doxycycline (10 mg/kg BID). However, a lower dose of doxycycline (5 mg/kg BID) did not eliminate *Wolbachia* from microfilariae by 56 days [10]. Our results suggest that by waiting 2 months to start melarsomine treatment, we are not risking potential damage to the vessels due to increasing worm size, and while the decrease in worm weight may be due to worm damage and *Wolbachia* depletion, the weight reduction observed in control groups cannot be adequately explained. Future work will be needed to assess *Wolbachia* levels in treated and untreated adult worms and the impact on worm weight.

There are some limitations to this study, some of which have been detailed above. First, we did not administer melarsomine. There exists the possibility that we would have observed worse lesions in dogs killed after melarsomine administration at day 30 versus day 60. Our study does, however, provide a baseline of lesion development after the administration of doxycycline and ivermectin. If we had administered melarsomine, then we could have observed differences in clinical signs, which would have provided additional “real-world” data that would help clinicians make a decision as to whether to start melarsomine administration at day 30 or 60. Another limitation was our inclusion of only one macrocyclic lactone, ivermectin. Many previous studies have used ivermectin with doxycycline to investigate the effectiveness of doxycycline in heartworm disease. To be able to extrapolate between studies, we chose to use ivermectin. It would be beneficial for us to repeat the above experiments with other MLs and formulations, as results could differ from the ones reported in this paper. Finally, we transplanted adult worms to study dogs rather than infecting them with third-stage infective larvae. We allowed worms 2.5 months to acclimate, which should have allowed them to have established an appropriate equilibrium with the host. This also allowed us to have control over the exact numbers of worms rather than the variability associated with larva-induced infections. There is the possibility that the worms could have reacted differently to the ivermectin/doxycycline regimen if they had gone through the natural migration pathway in the host. However, in larva-induced infection studies, there can be greater variability in controls, which could result in difficulty establishing statistical significance. With transplanted worms, this variability is reduced.

Another goal of our study was to examine the effect of doxycycline administration on liver enzyme parameters. In the current study, we did not note vomiting or other gastrointestinal signs that have been previously attributed to doxycycline administration [10, 12]. Furthermore, we noted only mildly elevated ALT in ten dogs, with moderately elevated ALT in two dogs. Five of these dogs had elevated ALT on day –1 and three others had high normal ALT on day –1 before doxycycline treatment. This indicates elevations in ALT are related to some other factor, possibly the heartworm infection itself or from the recent death of a heartworm. Dead and decomposing heartworms release foreign proteins into the circulation, which will filter through the liver where Kupffer cells phagocytize these materials. It has been shown that Kupffer cell activation can lead to liver injury in mice [20]. Three of the four remaining dogs developed mild elevations 2–4 weeks after doxycycline therapy and the one remaining dog only had a mildly elevated ALT at week 3 of therapy. These findings suggest that it is safe to use doxycycline prior to melarsomine. However, as we did not administer melarsomine in this study, side effects could have been observed later if we had continued the study through melarsomine treatment.

## Conclusions

No significant differences in histopathology are observed with or without the AHS-recommended 1-month wait period following doxycycline and ivermectin administration. Furthermore, reduced dosages of doxycycline demonstrated no worsening of pathology or change in efficacy in depleting worm weight. Mean individual worm weight did significantly decrease between 30 and 60 days post-treatment in all groups, including untreated controls, but this did not appear to correspond to doxycycline dosage. It is important to note that these results do not take melarsomine treatment into account, and future work will investigate the effects of this study’s treatment schemes on *Wolbachia* levels.

### Supplementary Information

**Additional file 1: Table S1:** Alanine aminotransferase (ALT) in dogs with elevated levels. Reference range = 17–115 U/l. Note that Group 6 was necropsied on day 30.

**Additional file 2: Table S2:** Weight of male and female worms at necropsy (g).

## Data Availability

The data that support the findings of this study are available from the corresponding author upon reasonable request.
